# Analysis of the predictive value of Th17/Treg cells and cytokines for the risk of infection after kidney transplantation

**DOI:** 10.3389/fimmu.2026.1701788

**Published:** 2026-04-30

**Authors:** Wei Li, Junyu Guo, Hongwei Yang, Boqian Wang

**Affiliations:** 1 Department of Intensive Care Unit, General Hospital of Northern Theater Command of Chinese People's Liberation Army (PLA), Shenyang, Liaoning, China; 2 Department of Pathology, General Hospital of Northern Theater Command of Chinese People's Liberation Army (PLA), Shenyang, Liaoning, China; 3 Department of Organ Transplant Center, General Hospital of Northern Theater Command of Chinese People's Liberation Army (PLA), Shenyang, Liaoning, China

**Keywords:** cytokines, independent risk factors, post-kidney transplantation infection, predictive value, Th17/Treg cells

## Abstract

**Objective:**

This study aimed to analyze the factors associated with infection after kidney transplantation and to explore the predictive value of Th17/Treg cells and cytokine levels for infection.

**Methods:**

A retrospective analysis was conducted on 200 patients who underwent kidney transplantation between May 2021 and January 2024. Patients were divided into an infection-free group (70 cases) and an infection group (130 cases) based on infection status within 1 year post-surgery. General clinical data were collected, and changes in Th17 and Treg cell ratios were detected using flow cytometry. Cytokine levels, including IL-17, IL-22, IL-10, and TGF-β, were measured using an enzyme-linked immunosorbent assay (ELISA) kit. Patients were divided into high- and low-expression groups based on the median serum Th17/Treg cell and cytokine levels, and the incidence of infection after kidney transplantation was compared between the two groups. Logistic regression analysis was used to identify independent risk factors for infection after kidney transplantation. Receiver operating characteristic (ROC) curve analysis was performed to assess the predictive value of Th17/Treg cell and cytokine levels for infection after kidney transplantation.

**Results:**

The infection group had a higher age and a higher proportion of preoperative hemodialysis compared to the non-infection group (p < 0.05). The infection group had higher Th17 cell proportions, Th17/Treg ratios, and serum IL-17 and IL-22 levels compared to the non-infection group, while the Treg cell proportions and serum IL-10 and TGF-β levels were lower in the infection group compared to the non-infection group (p < 0.001). High expression of Th17 cells and pro-inflammatory cytokines (IL-17 and IL-22) was associated with an increased risk of infection, while high expression of Treg cells and anti-inflammatory cytokines (IL-10 and TGF-β) was associated with a reduced risk of infection. Multivariate logistic regression analysis indicated that preoperative hemodialysis, elevated Th17/Treg ratio, elevated IL-17 and IL-22 levels, and reduced IL-10 and TGF-β levels were independent risk factors for infection after kidney transplantation (p < 0.05). ROC curve analysis indicated that the aforementioned indicators had high predictive value for infection within 1 year post-surgery, with the Area Under the Curve (AUC) of the Th17/Treg ratio reaching 0.927.

**Conclusion:**

Patients with post-transplant infections exhibit a significant Th17/Treg cell imbalance. Elevated Th17/Treg ratios, increased IL-17 and IL-22 levels, and decreased IL-10 and TGF-β levels are independent risk factors for post-transplant infections. These indicators also have high predictive value for post-transplant infection risk and may serve as potential biomarkers for early clinical assessment of infection risk.

## Introduction

With the development of society and the continuous advancement of medical technology, organ transplantation technology has become increasingly mature, providing effective treatment options for patients with end-stage organ failure (1). Among these, kidney transplantation stands out as a crucial treatment for end-stage renal failure, significantly improving patient survival rates and quality of life (2, 3). However, postoperative infection remains a common and severe complication. Patients who undergo kidney transplantation must take immunosuppressive medications long-term, which suppress the immune system and reduce the body’s ability to resist pathogens, thereby significantly increasing the risk of infection (4, 5). Postoperative infections not only impair the recovery of transplanted kidney function, prolong hospital stays, and increase medical costs but can also lead to graft failure or even threaten the patient’s life in severe cases (6, 7). Studies have indicated that approximately 70% of patients experience at least one infection within the first year post-transplant (8, 9). Therefore, early and accurate prediction of postoperative infection risk is crucial for implementing targeted preventive and therapeutic measures and improving patient outcomes.

Helper T lymphocytes 17 (Th17) and regulatory T cells (Treg) play a key role in immune regulation (10). Cytokines are small molecular proteins secreted by immune cells that play an important role in immune regulation and inflammatory responses. They form a complex intercellular communication network, binding to receptors on the surface of target cells to regulate the proliferation, differentiation, activation, and function of immune cells (11, 12). Th17 cells secrete pro-inflammatory factors (such as IL-17, IL-21, and IL-22), recruit neutrophils, and stimulate the secretion of inflammatory molecules that further participate in combating bacterial or fungal infections. In contrast, Treg cells produce anti-inflammatory cytokines IL-10 and TGF-β, which inhibit the activation and proliferation of effector T cells, thereby suppressing immune responses (13, 14). The balance between Th17 and Treg cells is crucial for maintaining immune stability in the body, and its disruption is associated with the development of various diseases, including rejection reactions and infections following organ transplantation (15).

However, the mechanisms underlying the roles of Th17/Treg cells and cytokines in post-transplant infections remain incompletely understood, and studies on their predictive value for post-transplant infection risk are limited. This study aimed to investigate the predictive value of Th17/Treg cells and related cytokines for post-transplant infection risk, providing a more comprehensive reference for the clinical prevention and treatment of post-transplant infections.

## Materials and methods

### Study population

This study was a retrospective analysis that included 200 patients who underwent kidney transplantation at our hospital between May 2021 and January 2024. Patients were divided into two groups based on whether they developed infection postoperatively: the non-infection group (70 cases) and the infection group (130 cases). The inclusion criteria were as follows: 1) eligible for kidney transplantation surgery and undergoing the procedure for the first time, 2) age between 18 and 70 years, and 3) complete clinical data available to meet research analysis requirements. The exclusion criteria were as follows: 1) concurrent organ dysfunction, 2) concurrent severe diseases (e.g., malignant tumors, severe cardiovascular disease, hepatic or renal insufficiency, and autoimmune diseases), 3) preoperative infection (upon verification, no patients in the original cohort had documented infections within the first 2 weeks prior to transplantation), and 4) patients who developed acute rejection postoperatively. This study strictly adhered to the provisions of the Declaration of Helsinki and was approved by the Medical Ethics Committee of our hospital.

### Infection diagnosis and monitoring criteria

Post-kidney transplant infection refers to any infectious disease that occurs in patients from the time of kidney transplant surgery until the 1-year follow-up period, including but not limited to the following: 1) Pulmonary infection: symptoms such as coughing, sputum production, and fever (body temperature ≥38.3 °C), chest imaging studies (X-ray or CT) showing pulmonary inflammatory infiltrates, and positive sputum culture or respiratory tract secretion pathogen detection or meeting clinical infection diagnostic criteria (e.g., community-acquired pneumonia or hospital-acquired pneumonia diagnostic criteria). 2) Urinary tract infections: symptoms such as frequent urination, urgency, or pain during urination, with urine routine tests showing white blood cell counts ≥5 per high-power field and urine culture showing bacterial colony counts ≥10^5^ CFU/mL. 3) Bloodstream infection: fever (body temperature ≥38.3 °C) or hypothermia (body temperature ≤36 °C), accompanied by chills or hypotension, with at least one blood culture positive for pathogens (excluding contaminating bacteria). 4) Surgical site infection: redness, swelling, pain, exudate, or purulent discharge at the surgical incision site, with positive culture of the discharge, or local tissue pathology examination indicating evidence of inflammation and infection. 5) Other infections: including abdominal infections, viral infections (e.g., cytomegalovirus and EB virus, confirmed by positive viral nucleic acid testing), and fungal infections (positive fungal culture or pathological examination), all diagnosed according to relevant clinical diagnostic and treatment guidelines.

All patients were hospitalized for observation for at least 2 weeks postoperatively and were followed up regularly until 1 year postoperatively. The follow-up frequency was as follows: once weekly within the first month postoperatively, every 2 weeks from 2 to 3 months postoperatively, monthly from 4 to 6 months postoperatively, and every 2 months from 7 to 12 months postoperatively. During follow-up, infection occurrence was monitored through clinical symptom inquiries, physical examinations, laboratory tests (complete blood count, inflammatory markers, pathogen detection, etc.), and imaging studies. All cases of infection were diagnosed by two or more kidney transplant specialists based on a comprehensive evaluation of the patient’s clinical symptoms, physical examination findings, laboratory tests, pathogen detection results (bacterial culture, viral nucleic acid testing, fungal culture, etc.), and imaging examination results, strictly adhering to the diagnostic criteria outlined in the “Clinical Practice Guidelines—Organ Transplantation” and relevant guidelines for the diagnosis and treatment of infectious diseases.

### Data collection

General clinical data were collected from all patients, including age, gender, body mass index (BMI), smoking history, alcohol consumption history, underlying conditions (history of hypertension and history of diabetes), and preoperative dialysis method (hemodialysis and peritoneal dialysis). Four milliliters of venous blood was collected from all study subjects prior to kidney transplantation. After allowing the blood sample to settle, samples were centrifuged, and the upper serum layer was aspirated and stored at −80 °C for subsequent testing. Flow cytometry was used to detect the proportions of Th17 cells and Treg cells in the patients’ peripheral blood, and the Th17/Treg ratio was calculated. Concurrently, the levels of IL-17, IL-22, IL-10, and TGF-β in the patients’ serum were measured using enzyme-linked immunosorbent assay (ELISA).

### Flow cytometry

In this study, flow cytometry was used to detect the proportion of Th17 cells (CD3+CD4+IL-17A) and Treg cells (CD4+CD25+CD127) in the peripheral blood of kidney transplant recipients prior to surgery. Four milliliters of venous blood was collected, treated with an anticoagulant, and centrifuged at 3,000 r/min for 20 minutes. The serum was separated and stored at −80 °C for future use. Peripheral blood mononuclear cells (PBMCs) were isolated using the Ficoll density gradient centrifugation method. For Th17 cell detection, PBMCs were seeded into a 24-well plate, stimulated with phorbol myristate acetate (PMA) and ionomycin (IO) for 1 hour, and then treated with a protein transport inhibitor Brefeldin A (BFA) and cultured at 37 °C for 4 hours. Subsequently, cells were labeled with CD3 Phycoerythrin (PE) and CD4 Fluorescein Isothiocyanate (FITC) antibodies, and incubated with IL-17A Allophycocyanin (APC) antibody after membrane disruption; the proportion was detected using flow cytometry. For Treg cell detection, PBMCs were directly labeled with CD4 (FITC), CD25 (PE), and CD127 (APC) antibodies; incubated in the dark for 20 minutes; washed; and resuspended. The proportion was detected using flow cytometry. The antibodies used included the following: PE anti-human CD3 (317308), FITC anti-human CD4 (344604), APC anti-human IL-17A (512334), PE anti-human CD25 (985802), and APC anti-human CD127 (351316), all purchased from BioLegend, San Diego, California; PMA (HY-18739), IO (HY-13434), and BFA (HY-16592) were purchased from MCE, Monmouth Junction, New Jersey.

### Enzyme-linked immunosorbent assay (ELISA)

Following the instructions in the ELISA kit manual, the levels of IL-17, IL-22, IL-10, and TGF-β in the serum were determined. The human IL-17 ELISA Kit (ab100556, detection range 15.63–1,000 pg/mL, sensitivity 10 pg/mL) was purchased from Abcam (Cambridge, UK); the human IL-22 ELISA kit (KIT13059, detection range 4.69–300 pg/mL, sensitivity 2.8 pg/mL) and the human IL-10 ELISA kit (KIT10947A, detection range 15.63–1,000 pg/mL, sensitivity 5.84 pg/mL) were purchased from SignalChem Biotech Inc. (Vancouver, Canada); human TGF-β ELISA Kit (EKH064, detection range 31.2–2,000 pg/mL, sensitivity 15.2 pg/mL) was purchased from Abixin (Shanghai) Biotechnology Co., Ltd. All experimental procedures were strictly conducted in accordance with the kit instructions.

### Statistical analysis

Data were analyzed and plotted using the GraphPad Prism 9.50 software (GraphPad Software Inc., San Diego, CA, USA) and SPSS 21.0 statistical software (SPSS, Inc., Chicago, IL, USA). The Shapiro–Wilk test was used to assess the normality of the data distribution. For normally distributed continuous variables, data were expressed as mean ± standard deviation (x ± s), and comparisons between groups were performed using the independent samples t-test. For non-normally distributed continuous variables, data were expressed as median (interquartile range) [M (Q1, Q3)], and comparisons were performed using the Mann–Whitney U test. Categorical data were expressed as counts and percentages (n, %). Comparisons between groups were performed using the chi-square test. Logistic regression analysis was used to identify independent risk factors for infection after kidney transplantation. Receiver operating characteristic (ROC) curve analysis was used to assess the predictive value of Th17/Treg cell and cytokine levels for infection after kidney transplantation. All p-values were obtained from two-sided tests, with p < 0.05 considered statistically significant.

## Results

### Comparison of general characteristics of the study population

A total of 200 patients who underwent kidney transplantation were included in this study. They were divided into an infection-free group (70 cases) and an infection group (130 cases) based on whether infection occurred within 1 year postoperatively. Statistical analysis of the general characteristics of all enrolled patients was conducted, as shown in **Table 1**. There were no significant differences between the two groups in terms of gender, BMI, smoking history, drinking history, hypertension history, or diabetes history (all p > 0.05). The mean age of the infection group was (52.25 ± 6.94) years, significantly higher than that of the non-infection group (46.67 ± 7.33) years (t = 5.313, p < 0.001). The proportion of patients in the infection group who underwent hemodialysis preoperatively was 66.92%, significantly higher than that of the non-infection group (45.71%), with a statistically significant difference (*χ*^2^ = 8.493, p = 0.003).

**Table 1:** General information on the enrolled population.

Project	Non-infection (N = 70)	Infection (N = 130)	t/*χ*^2^	P-value
Age (years)	46.67 ± 7.33	52.25 ± 6.94	5.313	<0.001
Gender (n, %)				
M	40 (57.14%)	78 (60.00%)	0.154	0.695
F	30 (42.86%)	52 (40.00%)		
BMI (kg/m^2^)	24.14 ± 1.39	24.51 ± 1.65	1.574	0.117
Smoking history (n, %)	22 (31.43%)	48 (36.92%)	0.604	0.437
Drinking history (n, %)	28 (40.00%)	56 (43.08%)	0.177	0.674
History of hypertension (n, %)	35 (50.00%)	76 (58.46%)	1.319	0.251
History of diabetes (n, %)	12 (17.14%)	26 (20.00%)	0.241	0.623
Preoperative dialysis method (n, %)				
Hemodialysis	32 (45.71%)	87 (66.92%)	8.493	0.003
Peritoneal dialysis	38 (54.29%)	43 (33.08%)		

Count data were expressed as counts and percentages and analyzed using the chi-square test. Continuous data that were normally distributed were expressed as mean ± standard deviation, and intergroup comparisons were performed using the independent samples t-test. p < 0.05 was considered statistically significant.

### Comparison of Th17/Treg cell levels between the two groups of patients

**Figure 1:**
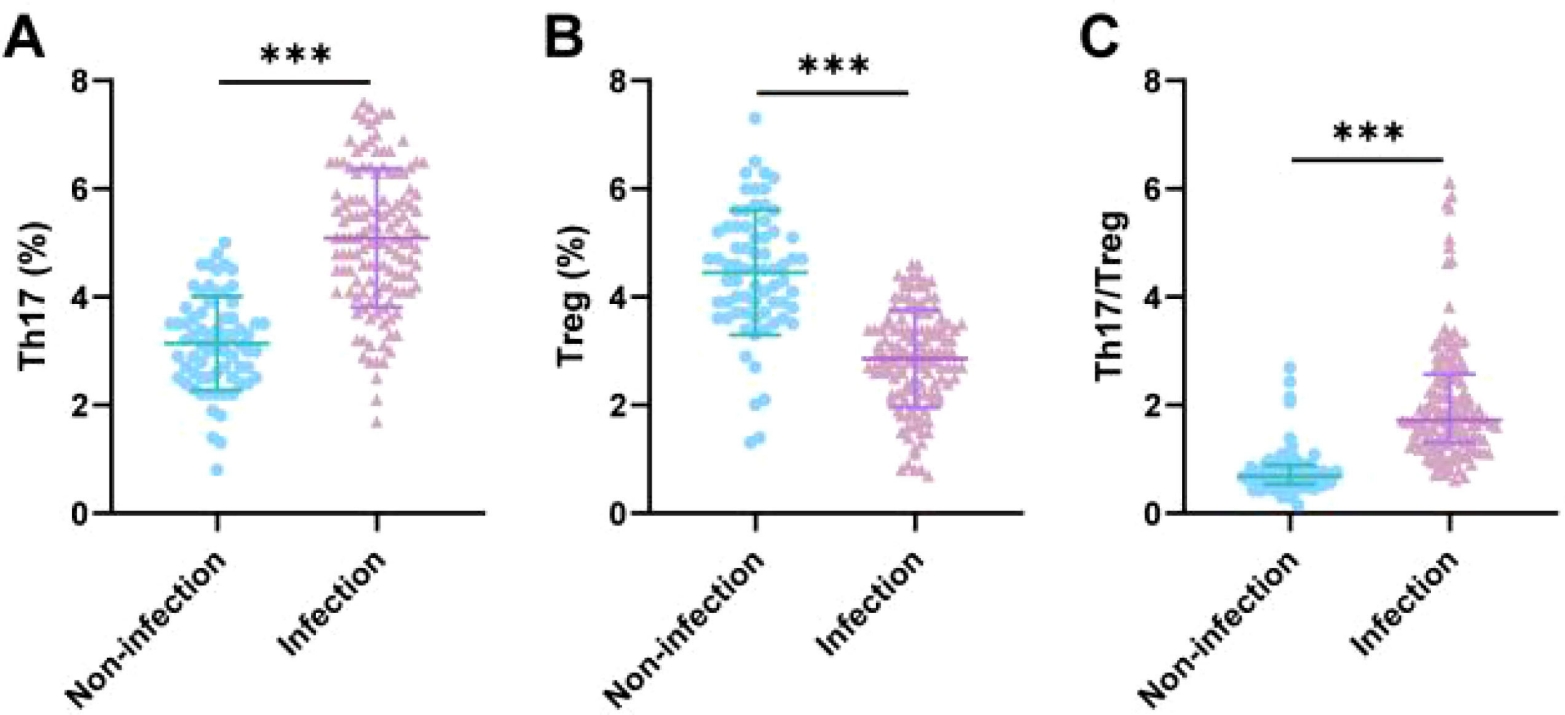
Comparison of Th17/Treg cell levels between the two groups of patients. **(A, B)** Th17 and Treg cell levels were detected by flow cytometry. **(C)** Calculation of the Th17/Treg ratio. The data in panels A and B follow a normal distribution and are expressed as mean ± standard deviation; an independent samples t-test was used. The data in panel C do not follow a normal distribution and are expressed as median (interquartile range); a Mann–Whitney U test was used. *** indicates p < 0.001.

It has been reported that the balance between Th17 and Treg cells is crucial for maintaining immune stability in the body and that an imbalance is associated with the onset and development of various diseases, including rejection reactions and infections after organ transplantation (15). Therefore, we used flow cytometry to detect changes in the ratio of Th17 and Treg cells. The results showed that, compared with the uninfected group, the infected group had a significantly higher proportion of Th17 cells, a significantly lower proportion of Treg cells, and a markedly increased Th17/Treg ratio (all p < 0.001) (**Figures 1A–C**). These results suggest that post-kidney transplantation infection patients exhibit a significant Th17/Treg cell imbalance, characterized by a predominance of pro-inflammatory Th17 cells and a reduction in anti-inflammatory Treg cells.

### Comparison of cytokine levels between the two groups of patients

**Figure 2:**
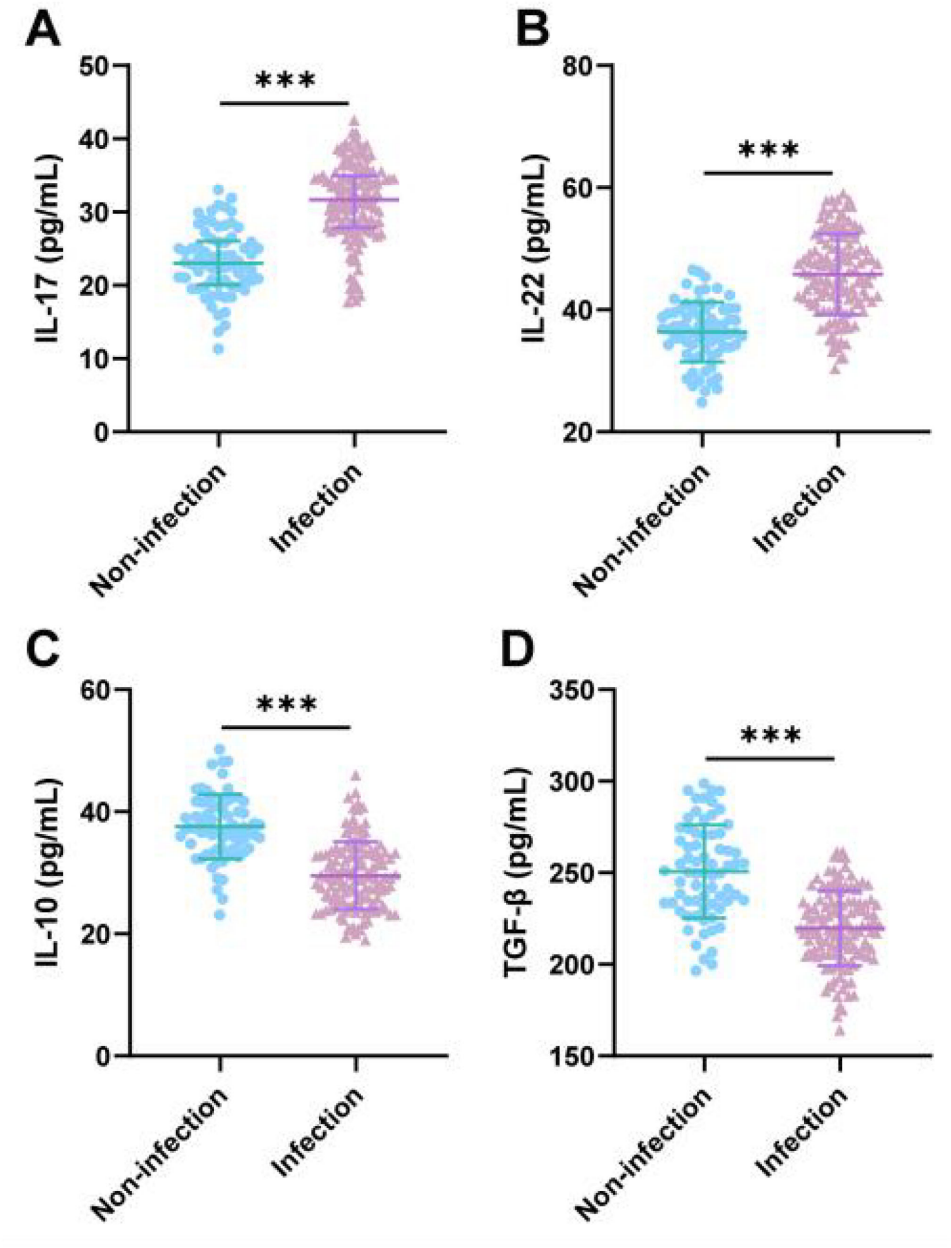
Comparison of cytokine levels between the two groups of patients. **(A–D)** ELISA kits were used to measure the levels of IL-17, IL-22, IL-10, and TGF-β in serum. In **(A)**, the data did not follow a normal distribution and were expressed as the median (interquartile range). The Mann–Whitney U test was used for analysis. The data in **(B–D)** follow a normal distribution and are expressed as the mean ± standard deviation. The independent samples t-test was used; *** indicates p < 0.001.

Cytokines are small molecular proteins secreted by immune cells that play an important role in immune regulation and inflammatory responses (11, 12). Further ELISA testing was conducted to measure the serum levels of Th17-related pro-inflammatory cytokines (IL-17 and IL-22) and Treg-related anti-inflammatory cytokines (IL-10 and TGF-β). The results showed that compared with those in the uninfected group, the levels of cytokines IL-17 and IL-22 in the serum of infected patients were significantly increased, while the levels of IL-10 and TGF-β were significantly decreased (all p < 0.001) (**Figures 2A–D**). These results suggest that pro-inflammatory cytokine secretion increases and anti-inflammatory cytokine secretion decreases in infected patients, further confirming the association between immune imbalance and postoperative infection.

### The relationship between Th17/Treg cells and cytokine levels, and the occurrence of infection after kidney transplantation

To further investigate the relationship between Th17/Treg cell and cytokine levels and the incidence of infection after kidney transplantation, we divided patients into high- and low-expression groups based on the median serum Th17/Treg cell and cytokine levels, and we compared the incidence of infection after kidney transplantation. The results showed that the incidence rates of infection in the high Th17 group, high Th17/Treg group, high IL-17 group, and high IL-22 group were 93.00%, 95.00%, 91.00%, and 92.00%, respectively, which were significantly higher than those of the corresponding low-expression groups (37.00%, 35.00%, 39.00%, and 38.00%, respectively). The infection rates in the high Treg group, high IL-10 group, and high TGF-β group were 39.00%, 40.00%, and 44.00%, respectively, significantly lower than those in the corresponding low-expression groups (91.00%, 90.00%, and 86.00%, respectively) (all p < 0.001) (**Table 2**). These results indicate that high expression of Th17 cells and pro-inflammatory cytokines (IL-17, IL-22) is associated with an increased risk of infection, while high expression of Treg cells and anti-inflammatory cytokines (IL-10 and TGF-β) is associated with a reduced risk of infection.

**Table 2:** Relationship between Th17/Treg cells and cytokine levels and the occurrence of infection after kidney transplantation.

Project	Not infected (N, %)	Infection (N, %)	*χ* ^2^	p
Low Th17 group (N = 100)	63 (63.00%)	37 (37.00%)	68.921	<0.001
High Th17 group (N = 100)	7 (7.00%)	93 (93.00%)		
Low Treg group (N = 100)	9 (9.00%)	91 (91.00%)	59.431	<0.001
High Treg group (N = 100)	61 (61.00%)	39 (39.00%)		
Low Th17/Treg group (N = 100)	65 (65.00%)	35 (35.00%)	79.121	<0.001
High Th17/Treg group (N = 100)	5 (5.00%)	95 (95.00%)		
Low IL-17 group (N = 100)	61 (61.00%)	39 (39.00%)	59.431	<0.001
High IL-17 group (N = 100)	9 (9.00%)	91 (91.00%)		
Low IL-22 group (N = 100)	62 (62.00%)	38 (38.00%)	64.091	<0.001
High IL-22 group (N = 100)	8 (8.00%)	92 (92.00%)		
Low IL-10 group (N = 100)	10 (10.00%)	90 (90.00%)	54.951	<0.001
High IL-10 group (N = 100)	60 (60.00%)	40 (40.00%)		
Low TGF-β group (N = 100)	14 (14.00%)	86 (86.00%)	38.771	<0.001
High TGF-β group (N = 100)	56 (56.00%)	44 (44.00%)		

### Multivariate logistic regression analysis of post-kidney transplant infection

To assess the factors influencing post-kidney transplant infection, with post-kidney transplant infection as the dependent variable (infection = 1, no infection = 0), we conducted a univariate logistic regression analysis using age, gender, BMI, smoking history, drinking history, hypertension history, diabetes history, and Th17 levels, Treg levels, Th17/Treg ratio, IL-17, IL-22, IL-10, and TGF-β as independent variables in a univariate logistic regression analysis. The results showed that age, preoperative dialysis method, Th17 levels, Treg levels, Th17/Treg ratio, IL-17, IL-22, IL-10, and TGF-β were significantly associated with infection within 1 year post-kidney transplantation (all p < 0.05) (**Table 3**). Subsequently, a multivariate logistic regression analysis was conducted using variables with p < 0.05 as independent variables (excluding Th17 and Treg levels to avoid multicollinearity). The results showed that preoperative dialysis method of hemodialysis (OR = 8.208, 95% CI: 1.198–56.236, p = 0.032), elevated Th17/Treg ratio (OR = 7.530, 95% CI: 1.978–28.674, p = 0.003), elevated IL-17 levels (OR = 1.353, 95% CI: 1.135–1.612, p < 0.001), elevated IL-22 levels (OR = 1.218, 95% CI: 1.036–1.431, p = 0.017), decreased IL-10 levels (OR = 0.815, 95% CI: 0.699–0.950, p = 0.009), and decreased TGF-β levels (OR = 0.950, 95% CI: 0.911–0.990, p = 0.014) were independent risk factors for postoperative infection (all p < 0.05) (**Table 3**).

**Table 3:** Logistic regression analysis of factors influencing the incidence of infection after kidney transplantation.

Project	Single-factor analysis			Multivariate analysis		
	P-value	OR value	95% CI	P-value	OR value	95% CI
Age (years)	<0.001	1.119	1.066–1.174	0.263	1.061	0.957–1.176
Gender (M = 1, F = 0)	0.695	1.125	0.624–2.028	–	–	–
BMI (kg/m^2^)	0.118	1.165	0.962–1.411	–	–	–
Smoking history (yes = 1, no = 0)	0.438	1.277	0.689–2.369	–	–	–
History of alcohol consumption (yes = 1, no = 0)	0.674	1.135	0.629–2.050	–	–	–
History of hypertension (yes = 1, no = 0)	0.252	1.407	0.785–2.524	–	–	–
History of diabetes (yes = 1, no = 0)	0.624	1.208	0.568–2.573	–	–	–
Preoperative dialysis method (hemodialysis = 1, peritoneal dialysis = 0)	0.004	2.403	1.325–4.358	0.032	8.208	1.198–56.236
Th17 (%)	<0.001	4.817	3.095–7.499	–	–	–
Treg (%)	<0.001	0.189	0.116–0.307	–	–	–
Th17/Treg	<0.001	41.396	13.721–124.889	0.003	7.530	1.978–28.674
IL-17 (pg/mL)	<0.001	1.343	1.237–1.458	<0.001	1.353	1.135–1.612
IL-22 (pg/mL)	<0.001	1.318	1.216–1.428	0.017	1.218	1.036–1.431
IL-10 (pg/mL)	<0.001	0.770	0.713–0.831	0.009	0.815	0.699–0.950
TGF-β (pg/mL)	<0.001	0.941	0.924–0.958	0.014	0.950	0.911–0.990

### The predictive value of Th17/Treg cells and cytokines for post-kidney transplantation infection

**Figure 3:**
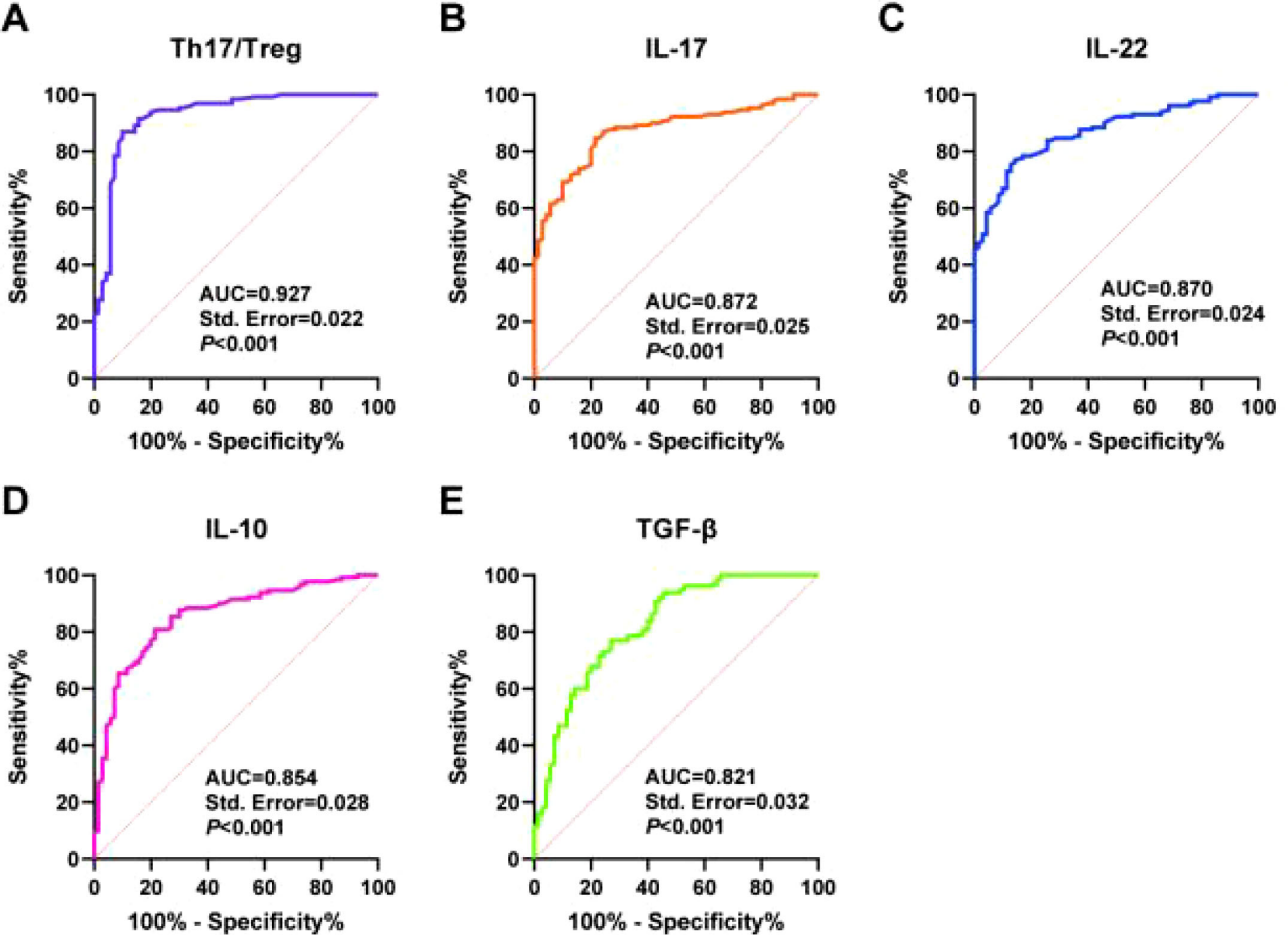
ROC analysis of the predictive value of Th17/Treg cells and cytokine levels for infection after kidney transplantation. Note: ROC curve analysis of Th17/Treg **(A)**, IL-17 **(B)**, IL-22 **(C)**, IL-10 **(D)**, and TGF-β **(E)** for predicting infection after kidney transplantation. ROC, receiver operating characteristic.

Based on the above results, we used ROC curves to assess the predictive value of Th17/Treg cell and cytokine levels for post-transplant infection in kidney transplant patients. The results showed that the AUC for Th17/Treg levels in predicting post-transplant infection in kidney transplant patients was 0.927, with a cutoff value of 1.10, a sensitivity of 86.92%, and a specificity of 90.00%. The AUC for serum IL-17 levels in predicting postoperative infection in kidney transplant patients was 0.872, with a cutoff value of 26.60, a sensitivity of 84.62%, and a specificity of 78.57%. The AUC for serum IL-22 levels in predicting postoperative infection in kidney transplant patients was 0.870, with a cutoff value of 41.05, a sensitivity of 76.92%, and a specificity of 85.71%. The AUC for serum IL-10 levels in predicting postoperative infection in kidney transplant patients was 0.854, with a cutoff value of 33.85, a sensitivity of 80.77%, and a specificity of 78.57%. The AUC for serum TGF-β levels in predicting postoperative infection in kidney transplant patients was 0.821, with a cutoff value of 234.70, a sensitivity of 76.92%, and a specificity of 72.86% (**Table 4**; **Figure 3**). These results indicate that Th17/Treg cell and cytokine levels have high predictive value for postoperative infection in kidney transplant patients.

**Table 4:** Predictive value of Th17/Treg cells and cytokine levels for infection after kidney transplantation.

Indicator	AUC	95% CI	Sensitivity (%)	Specificity (%)	Cutoff value
Th17/Treg	0.927	0.884–0.969	86.92	90.00	1.10
IL-17 (pg/mL)	0.872	0.823–0.920	84.62	78.57	26.60
IL-22 (pg/mL)	0.870	0.822–0.918	76.92	85.71	41.05
IL-10 (pg/mL)	0.854	0.799–0.908	80.77	78.57	33.85
TGF-β (pg/mL)	0.821	0.759–0.883	76.92	72.86	234.70

### Subgroup analyses stratified by infection type and immunosuppression protocol

To further explore the relationship between Th17/Treg imbalance and different infection types, we stratified the infection group by major infection categories (pulmonary, urinary tract, bloodstream, and surgical site infections). Due to the limited sample size in each subgroup, no statistically significant differences were observed in Th17/Treg ratios or cytokine levels across different infection types (**Supplementary Table 1**). Similarly, we analyzed the distribution of Th17/Treg ratios and infection rates across different immunosuppression protocols (tacrolimus-based, cyclosporine-based, and mTOR inhibitor-based regimens). While there was a trend toward higher Th17/Treg ratios in patients receiving tacrolimus-based immunosuppression, the differences did not reach statistical significance, likely due to the limited number of patients in each protocol subgroup (**Supplementary Table 2**). These exploratory analyses suggest that larger prospective studies with adequate statistical power are needed to detect potential differences in immune biomarker profiles across infection types and immunosuppression protocols.

## Discussion

Kidney transplantation is an important treatment option for patients with end-stage renal failure, significantly improving their quality of life. However, postoperative infection remains a critical complication threatening patient outcomes (16, 17). This study systematically analyzed clinical data from 200 kidney transplant patients, revealing a close association between Th17/Treg cell imbalance and abnormal levels of related cytokines with the risk of postoperative infection. This provides an important theoretical basis for early prediction and intervention of infection in clinical practice.

This study found that patients in the infection group exhibited significant Th17/Treg cell imbalance, characterized by elevated Th17 cell proportions and Th17/Treg ratios, while Treg cell proportions were reduced (p < 0.001). This phenomenon is highly consistent with the basic theory of immune regulation: Th17 cells, as a pro-inflammatory subset, recruit neutrophils by secreting cytokines such as IL-17 and IL-22, participating in inflammatory responses and pathogen clearance. Treg cells suppress excessive immune responses by secreting anti-inflammatory factors such as IL-10 and TGF-β, maintaining immune homeostasis (18–20). When this balance is disrupted, the body may suffer tissue damage due to excessive inflammation or increased susceptibility to infection due to insufficient immune suppression. In this study, the levels of pro-inflammatory factors IL-17 and IL-22 were significantly elevated, while the levels of anti-inflammatory factors IL-10 and TGF-β were significantly reduced (p < 0.001) in the infection group, further confirming that immune imbalance is one of the core mechanisms of postoperative infection.

From a clinical perspective, patients in the infection group were older (52.25 ± 6.94 vs. 46.67 ± 7.33 years) and had a higher proportion of preoperative hemodialysis (66.92% vs. 45.71%), suggesting that baseline immune status and preoperative treatment methods may influence postoperative immune balance. Immune function declines with age, leading to increased infection rates in the elderly (21), while long-term hemodialysis may further weaken immune reserves through repeated vascular access exposure and deteriorating nutritional status, creating a synergistic effect with Th17/Treg imbalance and increasing infection risk (22).

After grouping patients based on the median levels of cells and cytokines, it was found that the infection rates in the high Th17, high Th17/Treg, and high IL-17/IL-22 expression groups all exceeded 90%, while the infection rates in the high Treg and high IL-10/TGF-β groups were significantly reduced, suggesting a dose–response relationship between these markers and infection risk. Multivariate logistic regression analysis further confirmed that elevated Th17/Treg ratios, elevated IL-17 and IL-22 levels, and reduced IL-10 and TGF-β levels were independent risk factors for postoperative infection. Among these, preoperative hemodialysis, as a clinically modifiable factor, may be associated with increased risk due to immune activation, vascular endothelial damage, and microinflammatory states during dialysis (22, 23), suggesting that optimizing dialysis methods or shortening dialysis cycles may reduce the risk of postoperative infection in patients undergoing kidney transplantation. Among the immune markers, the Th17/Treg ratio had the highest OR value (7.530), and the ROC curve showed its optimal predictive value (AUC = 0.927, sensitivity 86.92%, specificity 90.00%), suggesting that this ratio can serve as a core indicator for assessing immune imbalance. IL-17, as a hallmark cytokine of Th17 cells, participates in the infection process by promoting inflammatory responses and tissue damage, consistent with previous studies on the pro-inflammatory role of IL-17 in post-transplant infections (24). The protective effects of IL-10 and TGF-β are manifested in inhibiting excessive immune responses and maintaining immune tolerance; their reduced levels directly weaken the body’s anti-inflammatory barriers (25, 26).

ROC curve analysis confirmed that the Th17/Treg ratio, IL-17, IL-22, IL-10, and TGF-β all have high predictive value for infection within 1 year post-surgery (AUC 0.821–0.927), with the Th17/Treg ratio demonstrating particularly outstanding diagnostic efficacy. This finding provides a basis for establishing a combined predictive model in clinical practice: by measuring these indicators preoperatively, patients can be categorized into high-risk and low-risk groups, enabling personalized prevention. For example, for high-risk patients with a Th17/Treg ratio >1.10, immunosuppressive therapy regimens can be adjusted in advance, infection prevention measures strengthened, or immunomodulatory therapy administered to reduce the incidence of infection.

From a mechanistic perspective, this study supports the hypothesis that “immune imbalance is the core driver of post-transplant infection”. Renal transplant patients, due to long-term use of immunosuppressive agents, experience suppressed Treg cell function while Th17 cells are relatively activated, leading to an imbalance in the pro-inflammatory–anti-inflammatory network (27, 28). This imbalance not only increases the risk of infection but may also cause sustained inflammatory damage to the transplanted kidney, forming a vicious cycle of “infection–inflammation–kidney damage”. Therefore, correcting Th17/Treg imbalance may serve as a dual-benefit therapeutic target, such as through Treg cell infusion or the use of IL-17 antagonists, which can both reduce infection risk and mitigate immune damage.

This study has several limitations that should be addressed in future research. First, the single-center retrospective design may introduce selection bias. Although the sample size of 200 patients is adequate for the primary analysis, a formal *a priori* power calculation was not performed. For detecting a 30% reduction in infection rates with 80% power, studies typically require approximately 206 patients per arm. The unequal group allocation in this study (70 non-infected vs. 130 infected patients) may limit the statistical power for certain subgroup analyses. Second, we performed exploratory stratified analyses by infection type and immunosuppression protocol; however, the limited sample sizes in each subgroup precluded the detection of statistically significant differences. These preliminary analyses suggest that larger multicenter studies are needed to definitively characterize infection type-specific immune signatures and the modulating effects of different immunosuppressive regimens on Th17/Treg balance. Third, this study only assessed preoperative immune biomarker levels without longitudinal monitoring at multiple postoperative time points. Dynamic assessment of Th17/Treg balance and cytokine profiles at various stages post-transplantation could provide crucial insights into the temporal evolution of immune dysfunction and its relationship with infection onset. Fourth, while this study focused on Th17/Treg balance and associated cytokines, other biomarkers such as immunoglobulin levels, complement factors, and CD4 counts have also shown predictive value in transplant infection risk assessment. A recent study demonstrated the utility of immunoglobulin profiling in predicting infectious complications (29). Comparative analyses incorporating multiple biomarker approaches would help establish the relative and complementary value of different predictive tools. Future research could be improved in several areas: first, conducting multicenter prospective cohort studies with adequate statistical power based on formal sample size calculations to enable robust subgroup analyses by infection type and immunosuppression protocol; second, establishing a dynamic monitoring system to track postoperative immune marker changes at multiple time points (e.g., at 1 week, 1 month, 3 months, 6 months, and 12 months post-transplantation) to identify infection-related warning thresholds and optimal intervention windows; third, performing head-to-head comparisons with other established biomarkers (immunoglobulin levels, complement factors, and CD4 counts) to determine the relative predictive performance and potential synergistic value of combined biomarker panels; and fourth, integrating basic research to explore intervention strategies for regulating Th17/Treg balance, such as cytokine-targeted therapy or immune cell infusion, to provide experimental evidence for clinical translation.

This study confirmed that patients with post-kidney transplant infections exhibit significant Th17/Treg cell imbalance and cytokine abnormalities. Preoperative hemodialysis, elevated Th17/Treg ratios, increased IL-17 and IL-22 levels, and decreased IL-10 and TGF-β levels are independent risk factors for postoperative infections. These indicators not only have high predictive value but also hold promise as targets for immune regulation, offering new insights for optimizing infection prevention and control strategies in kidney transplantation. In clinical practice, preoperative immune status assessment should be prioritized, with combined detection of the Th17/Treg ratio and related cytokines to achieve precise prediction and individualized management of infection risk.

## Data Availability

The original contributions presented in the study are included in the article/**Supplementary Material**. Further inquiries can be directed to the corresponding authors.
