# Supplementary material for: Analysis of the predictive value of Th17/Treg cells and cytokines for the risk of infection after kidney transplantation
Source: Front Immunol. 2026 Apr 30;17:1701788. doi: 10.3389/fimmu.2026.1701788 (PMC13171367; doi:10.3389/fimmu.2026.1701788)
Supplement: Supplementary file 1 [file DataSheet1.docx]

**（****Supplementary Materials）**

**Supplementary Table 1. Th17/Treg ratios and cytokine levels stratified by infection type**

| **Infection Type** | **N** | **Th17/Treg ratio** | **IL-17 (pg/mL)** | **IL-22 (pg/mL)** | **IL-10 (pg/mL)** | **TGF-β (pg/mL)** |
| --- | --- | --- | --- | --- | --- | --- |
| Pulmonary | 45 | 1.34 (1.15-1.58) | 28.45 ± 6.32 | 43.21 ± 8.15 | 31.24 ± 7.45 | 228.15 ± 38.42 |
| Urinary tract | 38 | 1.28 (1.12-1.52) | 27.18 ± 5.89 | 41.85 ± 7.92 | 32.15 ± 6.98 | 232.48 ± 35.67 |
| Bloodstream | 28 | 1.42 (1.18-1.65) | 29.32 ± 6.78 | 44.56 ± 8.48 | 30.12 ± 7.82 | 224.35 ± 41.23 |
| Surgical site | 19 | 1.31 (1.14-1.54) | 27.95 ± 6.15 | 42.38 ± 7.68 | 31.85 ± 7.12 | 230.72 ± 36.89 |
| P-value | - | 0.487 | 0.623 | 0.712 | 0.845 | 0.763 |

Data are expressed as median (interquartile range) for Th17/Treg ratio or mean ± standard deviation for cytokines. P-values were obtained by Kruskal-Wallis test (for Th17/Treg ratio) or one-way ANOVA (for cytokines). No statistically significant differences were observed across infection types, likely due to limited sample size in each subgroup. Note: Some patients had multiple concurrent infections and were categorized by their primary/most severe infection type.

**Supplementary Table 2. Th17/Treg ratios and infection rates stratified by immunosuppression protocol**

| **Immunosuppression Protocol** | **Total N** | **Infection N (%)** | **Non-infection N (%)** | **Th17/Treg ratio (Infection)** | **Th17/Treg ratio (Non-infection)** | **P-value** |
| --- | --- | --- | --- | --- | --- | --- |
| Tacrolimus-based | 142 | 94 (66.2%) | 48 (33.8%) | 1.35 (1.16-1.59) | 0.74 (0.58-0.89) | <0.001 |
| Cyclosporine-based | 38 | 24 (63.2%) | 14 (36.8%) | 1.28 (1.12-1.52) | 0.72 (0.56-0.87) | <0.001 |
| mTOR inhibitor-based | 20 | 12 (60.0%) | 8 (40.0%) | 1.24 (1.08-1.48) | 0.69 (0.54-0.84) | 0.002 |
| Between-protocol P-value | - | 0.834* | - | 0.512** | 0.678** | - |

Data for Th17/Treg ratios are expressed as median (interquartile range). *Chi-square test comparing infection rates across protocols. **Kruskal-Wallis test comparing Th17/Treg ratios across protocols within each group. Note: All protocols included mycophenolate mofetil and corticosteroids as baseline immunosuppression. The classification was based on the primary calcineurin inhibitor or mTOR inhibitor used. While infection rates and Th17/Treg ratios showed consistent patterns across all three protocol groups (with significant differences between infected and non-infected patients within each protocol), no significant differences were observed when comparing across different immunosuppression protocols, possibly due to the limited sample size in the cyclosporine and mTOR inhibitor groups.
